# Construction and validation of a prognostic nomogram for predicting cancer-specific survival in patients with intermediate and advanced colon cancer after receiving surgery and chemotherapy

**DOI:** 10.1007/s00432-023-05154-7

**Published:** 2023-07-17

**Authors:** Yiheng Shi, Xiaoting Wu, Wanxi Qu, Jiahao Tian, Xunlei Pang, Haohan Fan, Sujuan Fei, Bei Miao

**Affiliations:** 1 First Clinical Medical College, Xuzhou Medical University, Xuzhou, 221002 Jiangsu China; 2 Department of Gastroenterology, The Affiliated Hospital of Xuzhou Medical University, 99 West Huaihai Road, Xuzhou, 221002 Jiangsu China; 3 Institute of Gastroenterology, Xuzhou Medical University, 84 West Huaihai Road, Xuzhou, 221004 Jiangsu China; 4 Key Laboratory of Gastrointestinal Endoscopy, Xuzhou Medical University, Xuzhou, 221002 Jiangsu China

**Keywords:** Colon cancer, Nomogram, Cancer-specific survival, SEER, External validation

## Abstract

**Background:**

Existing predictive models often focus solely on overall survival (OS), neglecting the bias that other causes of death might introduce into survival rate predictions. To date, there is no strict predictive model established for cancer-specific survival (CSS) in patients with intermediate and advanced colon cancer after receiving surgery and chemotherapy.

**Methods:**

We extracted the data from the Surveillance, Epidemiology, and End Results (SEER) database on patients with stage-III and -IV colon cancer treated with surgery and chemotherapy between 2010 and 2015. The cancer-specific survival (CSS) was assessed using a competitive risk model, and the associated risk factors were identified via univariate and multivariate analyses. A nomogram predicting 1-, 3-, and 5-year CSS was constructed. The c-index, area under the curve (AUC), and calibration curve were adopted to assess the predictive performance of the model. Additionally, the model was externally validated.

**Results:**

A total of 18 risk factors were identified by univariate and multivariate analyses for constructing the nomogram. The AUC values of the nomogram for the 1-, 3-, and 5-year CSS prediction were 0.831, 0.842, and 0.848 in the training set; 0.842, 0.853, and 0.849 in the internal validation set; and 0.815, 0.823, and 0.839 in the external validation set. The C-index were 0.826 (se: 0.001), 0.836 (se: 0.002) and 0.763 (se: 0.013), respectively. Moreover, the calibration curve showed great calibration.

**Conclusion:**

The model we have constructed is of great accuracy and reliability, and can help physicians develop treatment and follow-up strategies that are beneficial to the survival of the patients.

**Supplementary Information:**

The online version contains supplementary material available at 10.1007/s00432-023-05154-7.

## Introduction

Colon cancer is one of the most prevalent gastrointestinal malignancies all around the world. It was reported that there were over 1.9 million patients diagnosed with colorectal cancer in 2020, and more than 935,000 patients died of it (Sung et al. 2020). Colon cancer is the fifth leading cause of cancer-induced death in China, and the number of the newly-diagnosed patients has surpassed that of gastric cancer (Cao et al. 2020, 2021). With the recent popularity of early screening for colon cancer and significant progress in the diagnosis and clinical management, the prognosis of colon cancer patients in China is significantly improved. However, these patients still have a poorer 5-year overall survival than the patients in developed countries such as Japan, the United States, Canada, and North Europe (Allemani et al. 2018).

Surgery combined with chemotherapy is the most important treatment for patients with intermediate and advanced colon cancer, which has been demonstrated to effectively improve the patients' prognosis and extend their survival (Labianca et al. 2010; Benson et al. 2018). During the follow-up process, some late-stage patients on surgery and chemotherapy were found to have survival time close to or even exceeding ten years. However, improvement in prognosis is typically accompanied by increased competitive risk. In addition to colon cancer-specific death, these patients could potentially die of other events such as cardiovascular diseases, chronic lower respiratory diseases, and accidents (Siegel et al. 2020). Compared with early-stage colon cancer, those at stages III or IV progress more rapidly, with higher-grade malignancy. These patients have a greater risk of recurrence and metastasis even after receiving surgery and chemotherapy, leading to a poorer prognosis (Snaebjornsson et al. 2017). In the clinical assessment of the prognosis of these patients, only all-cause mortality is often considered, while the impact of other competitive causes of death on patient prognosis is overlooked. This will obviously cause a significant predictive bias and reduce the predictive value of their true mortality rate. Therefore, from a clinical perspective, cancer-specific survival (CSS) can better reflect the basic characteristics of the tumor and the impact of treatment plans on survival. Additionally, identifying the CSS of patients can help develop treatment plans and follow-up strategies that are beneficial to the survival of patients. Moreover, studies have found that traditional survival analyses such as the Kaplan–Meier method and Cox regression tend to overestimate the risk of cancer-specific death in patients when competitive risks exist, as they treat competing events as independent outcomes, leading to the occurrence of competing risk bias (Cai et al. 2020; de Glas et al. 2016; Wang et al. 2022a). For patients with intermediate and advanced colon cancer who have achieved good survival benefits after systemic treatment, there is still a lack of a more comprehensive and accurate predictive model. To better guide clinical decision-making and provide patients with more accurate individualized risk assessments and prognostic management, competitive risk events should not be ignored. Both cancer and non-cancer mortality risks should be seriously considered by clinicians (Chesney et al. 2021). A competing risk model can correct the aforementioned bias (Walraven and Hawken 2016) and handle survival data with multiple outcome events, with its focus no longer limited to a single outcome indicator, and it has been widely applied clinically (Wang et al. 2022a, b; Lv et al. 2021a).

This study, based on the Surveillance, Epidemiology, and End Results (SEER) database, constructed a comprehensive competing risk nomogram model to predict the survival benefits of patients with intermediate and advanced colon cancer after receiving surgery and chemotherapy. The model was thoroughly validated using patient data from the Affiliated Hospital of Xuzhou Medical University.

## Methods

### Patients from SEER database

The data for modeling were collected from the SEER database. SEER is an open-access clinical database that contains cancer data of patients accounting for 30% of the U.S. population, covering 18 States (Doll et al. 2018). Data of the patients, who were diagnosed with stage-III and -IV colon cancer between 2010 and 2015 and had undergone surgery and chemotherapy, were collected. The detailed *inclusion criteria* were:

1. Patients were pathologically diagnosed with colon cancer, excluding the rectum (site recode, international classification of diseases for oncology ICD-O-3/WHO 2008).
2. The diagnosis was based on the criteria of AJCC Seventh Edition.
3. The patients had received surgery and chemotherapy.
4. Year of the diagnosis was from 2010 to 2015.

*Exclusion criteria*:

1. Expected survival time less than 1 month.
2. Stage-I and -II colon cancer patients diagnosed based on AJCC Seventh Edition.
3. Patients with incomplete or unclear demographic information, relevant clinical indicators, and survival status.

### External validation data and ethical statement

Data on 264 colon cancer patients at the Affiliated Hospital of Xuzhou Medical University from March 2014 to March 2018 were collected, according to the inclusion and exclusion criteria, to externally validate the model. The training set and internal validation data were obtained from the publicly available SEER database, and thus no additional informed consent was required. The external validation was conducted in accordance with the Declaration of Helsinki and approved by the Ethics Review Committee of Xuzhou Medical University, with the approval number: XYFY2023-KL004-01.

### Variable selection

Demographic information extracted from the SEER database includes Age recode with single ages and 100 + , Sex, Race recode (W, B, AI, API), and Marital status at diagnosis. Clinical indicators encompass Site recode ICD-O-3/WHO 2008, CS tumor size (2004–2015), Derived AJCC Stage Group, 7th ed (2010–2015), Grade, Surg Prim Site (1998 +), Regional nodes positive (1988 +), Scope Reg LN Sur (2003 +), Surg Oth Reg/Dis (2003 +), Radiation recode, CEA Pretreatment Interpretation Recode (2010 +), Perineural Invasion Recode (2010 +), Tumor Deposits Recode (2010 +), SEER Combined Mets at DX-bone (2010 +), SEER Combined Mets at DX-brain (2010 +), SEER Combined Mets at DX-liver (2010 +), SEER Combined Mets at DX-lung (2010 +), First malignant primary indicator, COD to site rec KM, Survival months, and SEER cause-specific death classification. The Lymph node ratio (LNR) is defined as Regional nodes positive/Scope Reg LN Sur. The outcome event is cancer-specific death, and the survival time is the time from the patient being diagnosed with intermediate and advanced colon cancer to death from colon cancer. The detailed selection process of patients is shown in Supplementary Table 1. For ease of subsequent research and analysis, we renamed and assigned values to the included indicators, as shown in Supplementary Table 2 and 3.

### Construction and validation of the competitive risk model

In this study, colon cancer-specific death was chosen as the outcome of interest, and deaths caused by other reasons were defined as competitive risk events. Patients still alive were classified as right-censored data. A competing risk model was used to predict the cumulative incidence function (CIF) of events. This model can handle survival data with multiple outcome events simultaneously, and can obtain more accurate predictions by calculating the CIF of each outcome event (Häggström et al. 2016). A total of 16,621 patients were randomly assigned in a 7:3 ratio into a training set (*n* = 11,634) and an internal validation set (*n* = 4987). The data of patients from our hospital were used as an external validation set (*n* = 264). Risk factors associated with the CSS of colon cancer patients were identified through univariate and multivariate analyses. We constructed a competitive risk model with these risk factors to predict the 1-year, 3-year, and 5-year CSS for patients with intermediate and advanced colon cancer. The c-index, receiver operator characteristic curve (ROC), area under the curve (AUC), and calibration curve were adopted to assess the predictive performance of the model. In addition, we compared the mortality predicted by the conventional survival analysis with that predicted by the competitive risk model.

### Statistical analyses

R studio and SPSS 26 software were adopted for statistical analyses. Categorical data were expressed as percentage and Chi-square test was applied to compare the differences between the groups. Continuous data in normal-distribution were described as mean ± standard deviation (SD). We calculated the cumulative incidence function (CIF) of each variable to avoid prediction bias in conventional analysis, and plotted CIF curve. Univariate analysis was performed using the Fine-Gray test, and variables with statistical significance were selected for multivariate analysis to identify CSS-associated independent risk factors. A competitive risk model-based nomogram was constructed using the Fine-Gray proportional hazards model. In addition, we compared the competitive risk model with conventional survival analysis in the predictive performance for the 1-year, 3-year, and 5-year CSS. A p value less than 0.05 would be considered statistically significant.

## Results

### Patients’ clinical characteristics

A total of 16,621 stage-III and -IV colon cancer patients were included, who had received surgery and chemotherapy. These patients were randomly assigned, in a 7:3 ratio, into a training set (*n* = 11,634) and an internal validation set (*n* = 4987). Patients at stage-III accounted for 67.6% (*n* = 11,242) and those at stage-IV accounted for 32.4% (*n* = 5379). For patients with distant metastases, most of them were liver metastasis (*n* = 3926, 23.6%). The incidence of tumor deposition (*n* = 4615, 27.7%) and perineural invasion (*n* = 3714, 22.3%) were low even in those patients. There were 8,703 CEA-positive patients (52.4%) and 7,091 CEA-normal patients (47.6%). There were 6165 patients who died of colon cancer, accounting for 78% of the total number of death, and 1,735 patients died of other reasons, accounting for 22%. The external validation set included information from 264 patients. Among these patients, 184 (70%) were stage-III colon cancer patients and 80 (30%) were stage-IV colon cancer patients. By the end of the follow-up, 123 patients (45.4%) were still alive, 124 died specifically of colon cancer, accounting for 87.9% of the total number of death, and 17 died of other reasons, accounting for 12.1%. Clinical information and demographical characteristics of the patients are shown in Table 1.

**Table 1:** Clinical information and demographical characteristics of the patients in the training, internal validation, and external validation sets

	SEER database			Our own database	
	Training set (*N* = 11,634)	Internal validation set (*N* = 4987)	All (*N* = 16,621)	External validation set (*N* = 264)	*P* value
Age					
	62.27 ± 12.81	62.01 ± 12.91		59.03 ± 12.25	< 0.01
Sex					
Male	5956 (0.51)	2573 (0.52)	8529 (0.51)	151 (0.57)	0.148
Female	5678 (0.49)	2414 (0.48)	8092 (0.49)	113 (0.43)	
Race					
White	8815 (0.76)	3787 (0.76)	12,602 (0.76)	NA	0.703
Black	1641 (0.14)	715 (0.14)	2356 (0.14)	NA	
Other	1178 (0.1)	485 (0.1)	1663 (0.1)	NA	
Marital_status					
Married	7024 (0.6)	2973 (0.6)	9997 (0.6)	233 (0.88)	< 0.01
Single	2021 (0.17)	846 (0.17)	2867 (0.17)	4 (0.02)	
Other	2589 (0.22)	1168 (0.23)	3757 (0.23)	27 (0.1)	
Tumor_site					
Ascending Colon	2185 (0.19)	956 (0.19)	3141 (0.19)	53 (0.2)	< 0.01
Cecum	2956 (0.25)	1296 (0.26)	4252 (0.26)	38 (0.14)	
Descending Colon	820 (0.07)	347 (0.07)	1167 (0.07)	23 (0.09)	
Hepatic Flexure	539 (0.05)	233 (0.05)	772 (0.05)	22 (0.08)	
Sigmoid Colon	3616 (0.31)	1511 (0.3)	5127 (0.31)	104 (0.39)	
Splenic Flexure	454 (0.04)	189 (0.04)	643 (0.04)	13 (0.05)	
Transverse Colon	1064 (0.09)	455 (0.09)	1519 (0.09)	11 (0.04)	
Tumor_grade					
I/II	8390 (0.72)	3615 (0.72)	12,005 (0.72)	150 (0.57)	< 0.01
III/IVI	3244 (0.28)	1372 (0.28)	4616 (0.28)	114 (0.43)	
Tumor_stage					
III	7865 (0.68)	3377 (0.68)	11,242 (0.68)	184 (0.7)	0.769
IV	3769 (0.32)	1610 (0.32)	5379 (0.32)	80 (0.3)	
T_stage					
T1–2	1074 (0.09)	466 (0.09)	1540 (0.09)	3 (0.01)	< 0.01
T3	7184 (0.62)	3045 (0.61)	10,229 (0.62)	121 (0.46)	
T4	3376 (0.29)	1476 (0.3)	4852 (0.29)	140 (0.53)	
N_stage					
NO/N1	7064 (0.61)	2990 (0.6)	10,054 (0.6)	167 (0.63)	0.431
N2	4570 (0.39)	1997 (0.4)	6567 (0.4)	97 (0.37)	
Surg_type					
Partial colectomy	4464 (0.38)	1891 (0.38)	6355 (0.38)	75 (0.28)	0.011
Subtotal colectomy	6751 (0.58)	2891 (0.58)	9642 (0.58)	183 (0.7)	
Total colectomy	237 (0.02)	113 (0.02)	350 (0.02)	3 (0.01)	
Other	182 (0.02)	92 (0.02)	274 (0.02)	3 (0.01)	
Lymphadenectomy					
0	114 (0.01)	62 (0.01)	176 (0.01)	4 (0.02)	0.584
1–3	135 (0.01)	56 (0.01)	191 (0.01)	3 (0.01)	
> 4	11,385 (0.98)	4869 (0.98)	16,254 (0.98)	257 (0.97)	
Metastasectomy					
No	10,011 (0.86)	4296 (0.86)	14,307 (0.86)	240 (0.91)	0.078
Yes	1623 (0.14)	691 (0.14)	2314 (0.14)	24 (0.09)	
Radiation					
No/Unknown	11,269 (0.97)	4849 (0.97)	16,118 (0.97)	253 (0.96)	0.251
Yes	365 (0.03)	138 (0.03)	503 (0.03)	11 (0.04)	
CEA					
Negative/normal	5531 (0.48)	2387 (0.48)	7918 (0.48)	122 (0.46)	0.836
Positive/elevated	6103 (0.52)	2600 (0.52)	8703 (0.52)	142 (0.54)	
Perineural_Invasion					
Not identified/present	9050 (0.78)	3857 (0.77)	12,907 (0.78)	191 (0.72)	0.110
Identified/present	2584 (0.22)	1130 (0.23)	3714 (0.22)	73 (0.28)	
Tumor_Deposits					
No	8429 (0.72)	3577 (0.72)	12,006 (0.72)	180 (0.68)	0.219
Yes	3205 (0.28)	1410 (0.28)	4615 (0.28)	84 (0.32)	
LNR	0.22 ± 0.22	0.23 ± 0.23		0.23 ± 0.24	0.418
Bone_metastasis					
No	11,549 (0.99)	4950 (0.99)	16,499 (0.99)	259 (0.98)	0.096
Yes	85 (0.01)	37 (0.01)	122 (0.01)	5 (0.02)	
Brain_metastasis					
No	11,604 (1)	4979 (1)	16,583 (1)	264 (1)	0.353
Yes	30 (0)	8 (0)	38 (0)	0 (0)	
Liver_metastasis					
No	8903 (0.77)	3792 (0.76)	12,695 (0.76)	214 (0.81)	0.163
Yes	2731 (0.23)	1195 (0.24)	3926 (0.24)	50 (0.19)	
Lung_metastasis					
No	11,093 (0.95)	4744 (0.95)	15,837 (0.95)	247 (0.94)	0.352
Yes	541 (0.05)	243 (0.05)	784 (0.05)	17 (0.06)	
Tumor_size	51.47 ± 30.10	52.60 ± 45.67		54.30 ± 20.29	0.095
First_malignant					
No	1576 (0.14)	687 (0.14)	2263 (0.14)	NA	0.694
Yes	10,058 (0.86)	4300 (0.86)	14,358 (0.86)	NA	
Total_nunmber	0.00 ± 0.07	0.01 ± 0.08		NA	0.178

### Nomogram construction based on the competitive risk model

**Fig. 1:**
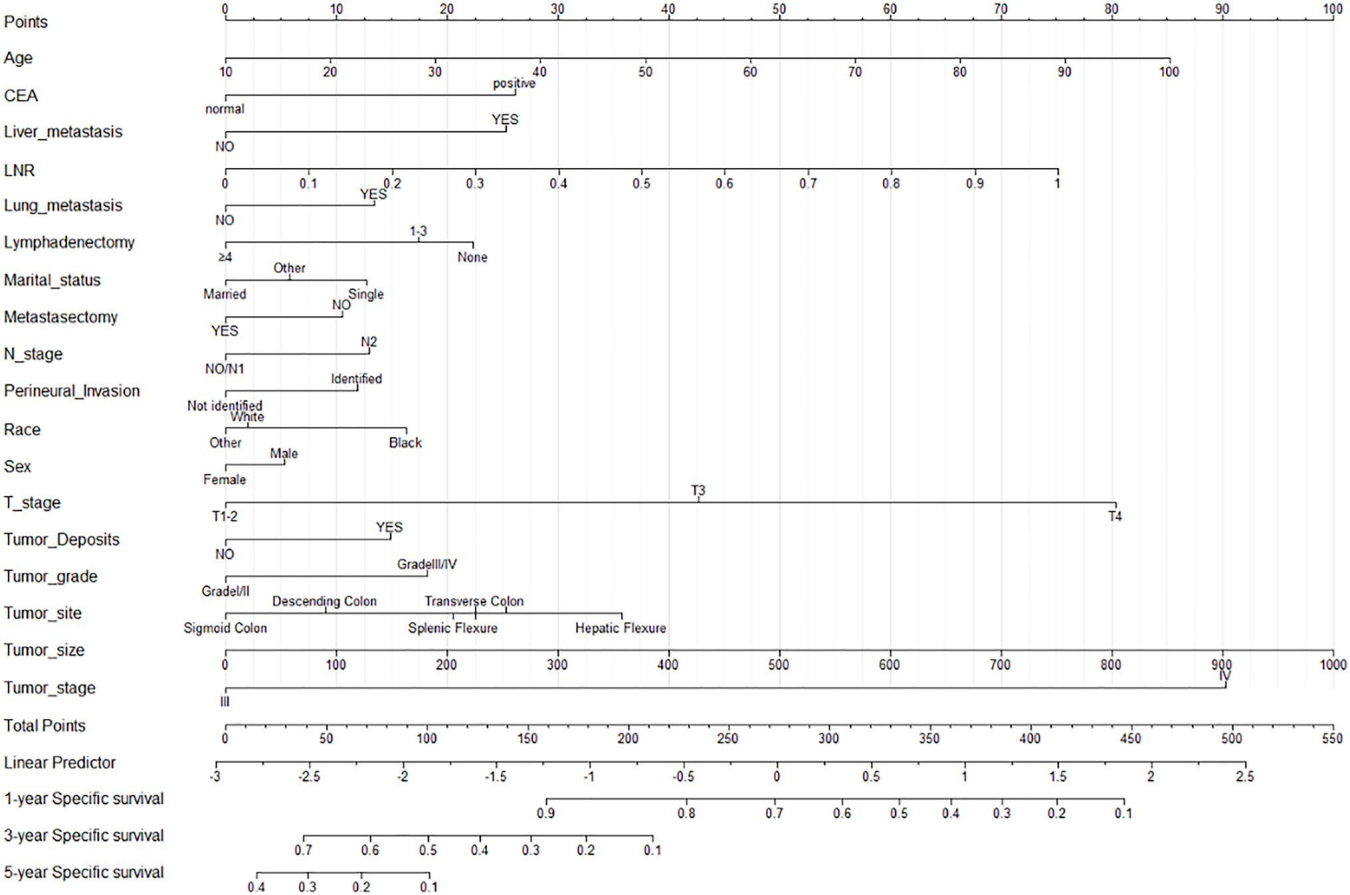
1-year, 3-year, and 5-year CSS-prediction nomogram

**Fig. 2:**
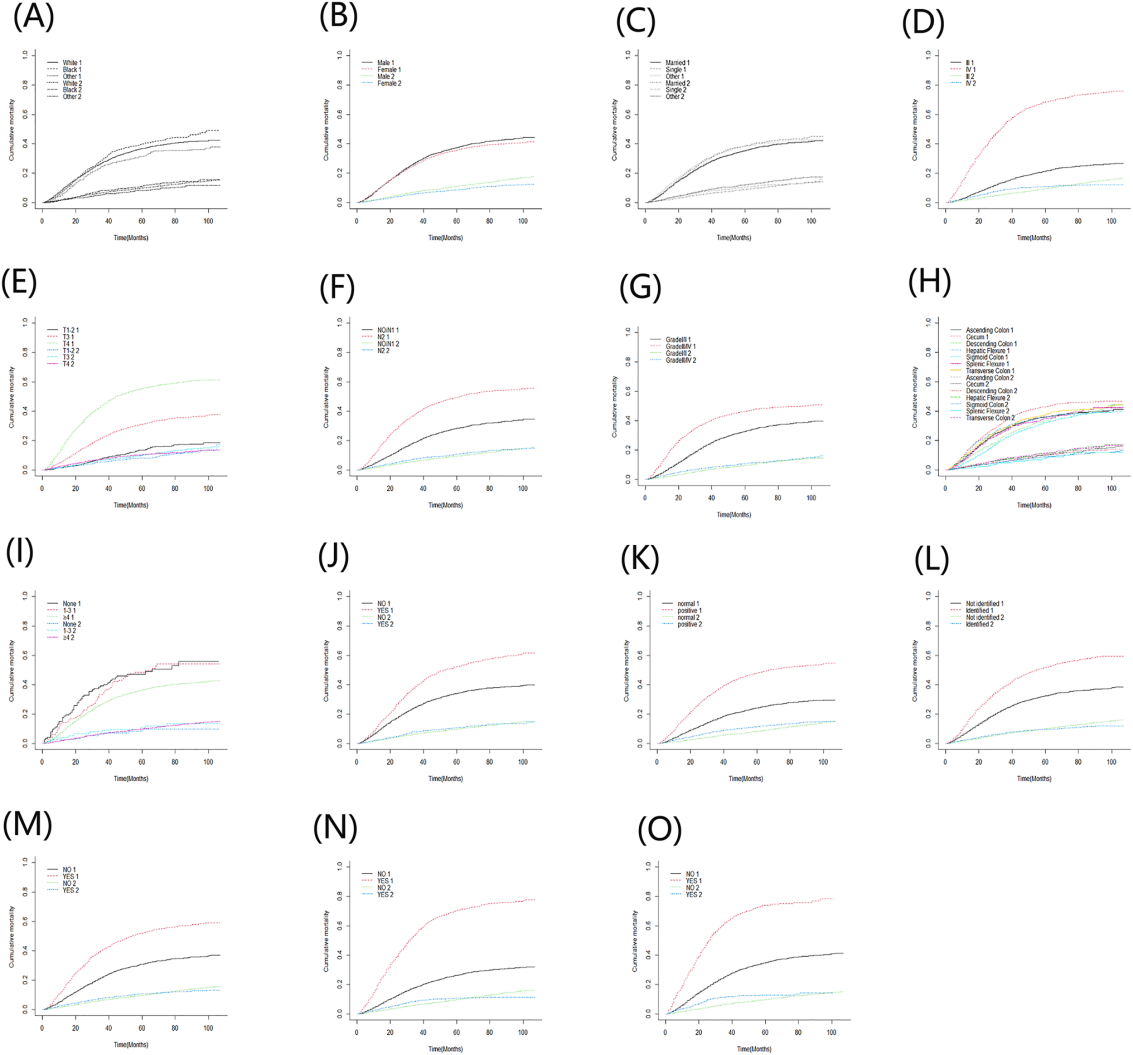
Cumulative cancer-specific mortality of each independent risk factor (CIF) **A** race; **B** sex; **C** marital status; **D** tumor stage; **E** T stage; **F** N stage; **G** tumor grade; **H** tumor site; **I** lymphadenectomy; **J** metastasectomy; **K** CEA; **L** perineural invasion; **M** tumor deposits; **N** liver metastasis; **O** lung metastasis; “1” represented cancer-specific death, and “2” represented death from other causes

Univariate analysis showed that Age, Sex, Race, Marital status, Tumor site, grade, stage, T stage, N stage, Surgery type, Lymphadenectomy, Metastasectomy, Radiation, CEA, Perineural Invasion, Tumor Deposits, Tumor size, LNR, Bone metastasis, Brain metastasis, Liver metastasis, and Lung metastasis are independent risk factors for colon cancer-specific death. Multivariate analysis of the above risk factors showed: age (HR 1.01,95% CI 1–1.01), Marital status (married as a reference; single: HR 1.11, 95% CI 1.02–1.21), Sex (male as a reference; female: HR 0.93, 95% CI 0.87–1), Race (white as a reference; black: HR 1.17,95% CI 1.07–1.28), Tumor grade (grade I/II as a reference; grade III/IV: HR 1.26, 95% CI 1.17–1.36), Tumor stage (stage III as a reference; stage IV: HR 2.78 2.49–2.39), Tumor size (HR 1, 95% CI 1–1), Tumor site (Ascending Colon as a reference; Hepatic Flexure (HR 1.18,95% CI 1–1.4; Sigmoid Colon: HR 0.78,95% CI 0.7–0.88), T stage (T1-2 as a reference; T3: HR 1.51, 95% CI 1.29–1.77; T4: HR 2.31, 95% CI 1.96–2.73), N stage (N0/N1 as a reference; N2: HR 1.11, 95% CI 1.01–1.21), Lymphadenectomy (0 as a reference; > 4: HR 0.7, 95% CI 0.52–0.94), Metastasectomy (NO as a reference; YES: HR 0.85, 95% CI 0.78–0.93), LNR (HR 2.9,95% CI 2.41–3.47), CEA (negative/normal as a reference; positive/elevated: HR 1.33, 95% CI 1.23–1.43), Perineural Invasion (Not identified/present as a reference, Identified/present: HR 1.14, 95% CI 1.06–1.23), Tumor Deposits (NO as a reference; YES: HR 1.18, 95% CI 1.1–1.27), Lung metastasis (NO as a reference; YES: HR 1.19, 95% CI 1.05–1.35), and Liver metastasis (NO as a reference; YES: HR 1.4, 95% CI 1.27–1.56) are independent risk factors for colon cancer-specific death (Table 2). Based on the results of multivariate analysis, a nomogram based on competing risk model was constructed to predict the 1-year, 3-year, and 5-year CSS of stage-III and -IV colon cancer patients after surgery and chemotherapy (Fig. 1). According to the competing risk model, we calculated the CIF for each independent risk factor affecting patient prognosis, as shown in Fig. 2, in which the number 1 indicates cancer-specific death and number 2 indicates death from other causes.

**Table 2:** Univariate and multivariate analysis of CSS in the training set

	Univariate analysis		Multivariate analysis	
	HR (95% CI)	*Z* (*P*)	HR (95% CI)	*Z* (*P*)
Age				
	1.01 (1–1.01)	< 0.001	1.01 (1.01–1.01)	< 0.001
Sex				
Male	Ref	NA	Ref	NA
Female	0.94 (0.88–0.99)	0.0330	0.93 (0.87–1)	0.0410
Race				
White	Ref	NA	Ref	NA
Black	1.12 (1.03–1.21)	0.0092	1.17 (1.07–1.28)	< 0.001
Other	0.84 (0.76–0.94)	0.0018	0.96 (0.86–1.08)	0.5400
Marital_status				
Married	Ref	NA	Ref	NA
Single	1.11 (1.02–1.2)	0.0130	1.11 (1.02–1.21)	0.0200
Other	1.09 (1.01–1.17)	0.0230	1.06 (0.98–1.15)	0.1700
Tumor_site				
Ascending colon	Ref	NA	Ref	NA
Cecum	1.26 (1.15–1.37)	< 0.001	1.05 (0.95–1.15)	0.3600
Descending colon	0.93 (0.81–1.06)	0.2800	0.89 (0.77–1.02)	0.0970
Hepatic flexure	1.03 (0.88–1.21)	0.7400	1.18 (1–1.4)	0.0450
Sigmoid colon	0.86 (0.78–0.94)	0.0007	0.78 (0.7–0.88)	< 0.001
Splenic flexure	0.99 (0.84–1.17)	0.9000	0.94 (0.79–1.13)	0.5200
Transverse colon	1.07 (0.95–1.21)	0.2600	1.02 (0.89–1.16)	0.8200
Tumor_grade				
I/II	Ref	NA	Ref	NA
III/IV	1.64 (1.54–1.75)	< 0.001	1.26 (1.17–1.36)	< 0.001
Tumor_stage				
III	Ref	NA	Ref	NA
IV	4.76 (4.48–5.06)	< 0.001	2.78 (2.49–3.09)	< 0.001
T_stage				
T1–2	Ref	NA	Ref	NA
T3	2.52 (2.14–2.96)	< 0.001	1.51 (1.29–1.77)	< 0.001
T4	5.53 (4.7–6.51)	< 0.001	2.31 (1.96–2.73)	< 0.001
N_stage				
NO/N1	Ref	NA	Ref	NA
N2	2.09 (1.97–2.21)	< 0.001	1.11 (1.01–1.21)	0.0240
Surg_type				
Partial colectomy	Ref	NA	Ref	NA
Subtotal colectomy	1.18 (1.11–1.25)	< 0.001	0.97 (0.9–1.06)	0.5500
Total colectomy	1.17 (0.95–1.44)	0.1400	1.01 (0.8–1.29)	0.9100
Other	1.49 (1.19–1.85)	0.0004	1.2 (0.95–1.51)	0.1400
Lymphadenectomy				
0	Ref	NA	Ref	NA
1–3	0.92 (0.65–1.32)	0.6600	0.82 (0.55–1.23)	0.3400
> 4	0.64 (0.49–0.84)	0.0014	0.7 (0.52–0.94)	0.0160
Metastasectomy				
No	Ref	NA	Ref	NA
Yes	1.76 (1.64–1.89)	< 0.001	0.85 (0.78–0.93)	< 0.001
Radiation				
No/Unknown	Ref	NA	Ref	NA
Yes	1.46 (1.25–1.69)	< 0.001	1.15 (0.97–1.37)	0.1100
CEA				
Negative/normal	Ref	NA	Ref	NA
Positive/elevated	2.34 (2.19–2.49)	< 0.001	1.33 (1.23–1.43)	< 0.001
Perineural_Invasion				
Not identified/present	Ref	NA	Ref	NA
Identified/present	1.87 (1.75–1.99)	< 0.001	1.14 (1.06–1.23)	< 0.001
Tumor_Deposits				
No	Ref	NA	Ref	NA
Yes	2 (1.88–2.13)	< 0.001	1.18 (1.1–1.27)	< 0.001
LNR	7.7 (6.84–8.67)	< 0.001	2.9 (2.41–3.47)	< 0.001
Bone_metastasis				
No	Ref	NA	Ref	NA
Yes	4.71 (3.56–6.23)	< 0.001	1.4 (0.98–1.99)	0.0620
Brain_metastasis				
No	Ref	NA	Ref	NA
Yes	3.03 (1.7–5.39)	< 0.001	1.32 (0.76–2.3)	0.3200
Liver_metastasis				
No	Ref	NA	Ref	NA
Yes	3.9 (3.68–4.15)	< 0.001	1.4 (1.27–1.56)	< 0.001
Lung_metastasis				
No	Ref	NA	Ref	NA
Yes	3.13 (2.81–3.48)	< 0.001	1.19 (1.05–1.35)	0.0065
Tumor_size	1 (1–1)	< 0.001	1 (1–1)	< 0.001
First_malignant				
No	Ref	NA	Ref	NA
Yes	1 (0.92–1.1)	0.9500	Ref	NA
Total_nunmber	0.79 (0.46–1.36)	0.4000	NA	NA

### Nomogram Verification

**Fig. 3:**
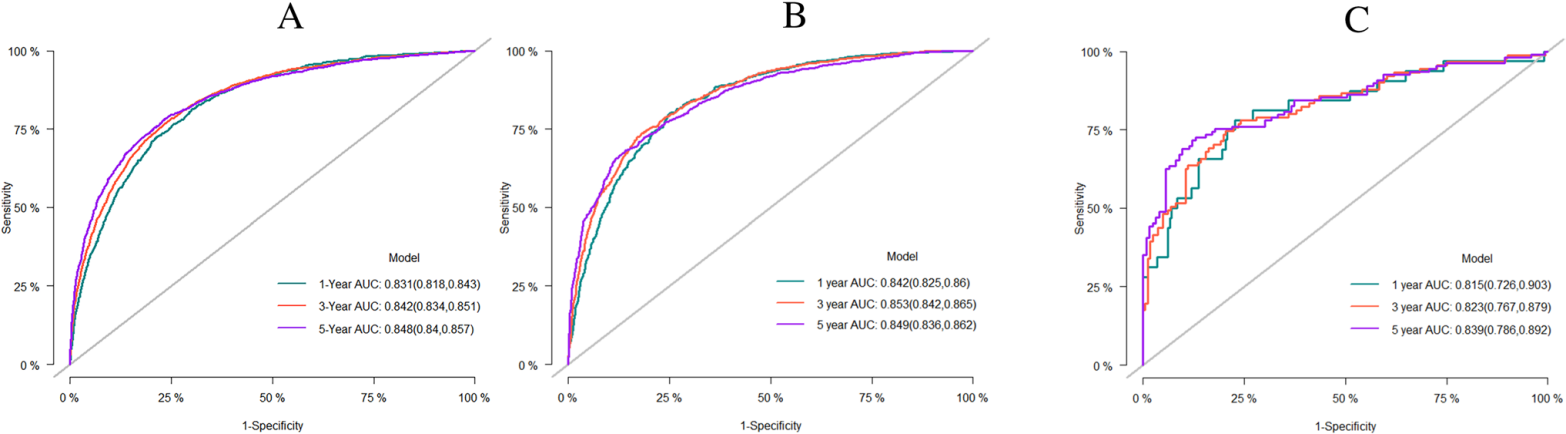
ROC of the competitive risk nomogram predicting the 1-year, 3-year, and 5-year CSS; **A** training set, **B** internal validation set, **C** external validation set; ROC, receiver operating characteristic; CSS, cancer-specific survival; AUC, area under ROC curve

**Fig. 4:**
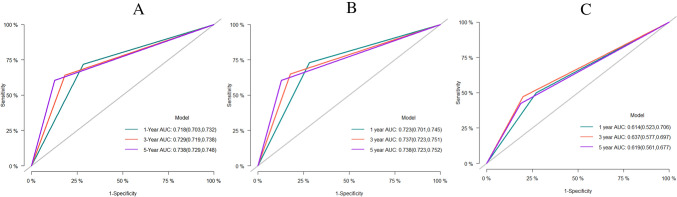
ROC of the AJCC-TNM staging system predicting the 1-year, 3-year, and 5-year CSS; **A** training set, **B** internal validation set, **C** external validation set

**Fig. 5:**
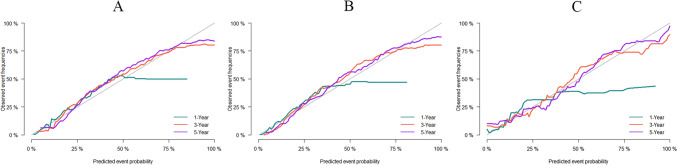
Calibration curve of the competitive risk nomogram predicting the 1-year, 3-year, and 5-year CSS; **A** training set, **B** internal validation set, **C** external validation set

This study applied c-index, ROC, and AUC to assess the predictive accuracy of the nomogram, and tested its calibration through providing a calibration curve. The c-index of the nomogram in the training set was 0.826 (se:0.001), and the AUC predicting the 1-year, 3-year, and 5-year CSS was 0.831 (95% CI 0.818–0.843), 0.842 (95% CI 0.834–0.851), and 0.848 (95% CI 0.840–0.857), respectively (Fig. 3A). This suggested the model was of considerably accurate predictive performance in the training set. The c-index in the internal validation set and external validation set was 0.836 (se: 0.002) and 0.763 (se: 0.013), respectively. The AUC predicting the 1-year, 3-year, and 5-year CSS was 0.842 (95% CI 0.825–0.860), 0.853 (95% CI 0.842–0.865), and 0.849 (95% CI 0.836- 0.862), respectively, in the internal validation set (Fig. 3B), and were 0.815 (95% CI 0.726–0.903), 0.823 (95% CI 0.767–0.879), 0.839 (95% CI 0.786–0.892), respectively, in the external validation set (Fig. 3C). This indicated the model was of great predictive value in both the internal and external validation, with high reliability. We further compared it with the conventional AJCC-TNM staging model. The AUC of AJCC-TNM staging model in predicting the 1-year, 3-year, and 5-year CSS was 0.718 (95% CI 0.703–0.732), 0.729 (95% CI 0.719–0.738), and 0.738 (95% CI 0.729–0.748), respectively, in the training set (Fig. 4A), were 0.723 (95% CI 0.701–0.745), 0.737 (95% CI 0.723–0.751), and 0.738 (95% CI 0.723–0.752), respectively, in the internal validation set (Fig. 4B), and were 0.614 (95% CI 0.523–0.706), 0.637(95% CI 0.577–0.697), 0.619 (95% CI 0.561–0.677), respectively, in the external validation set (Fig. 4C). The results firmly supported that the competitive risk model that we constructed was more effective than the conventional AJCC-TNM staging model. There was a good calibration between the predicted risk and the actual risk in both the training set and the validation set (Fig. 5A-C).

### Risk stratification

**Fig. 6:**
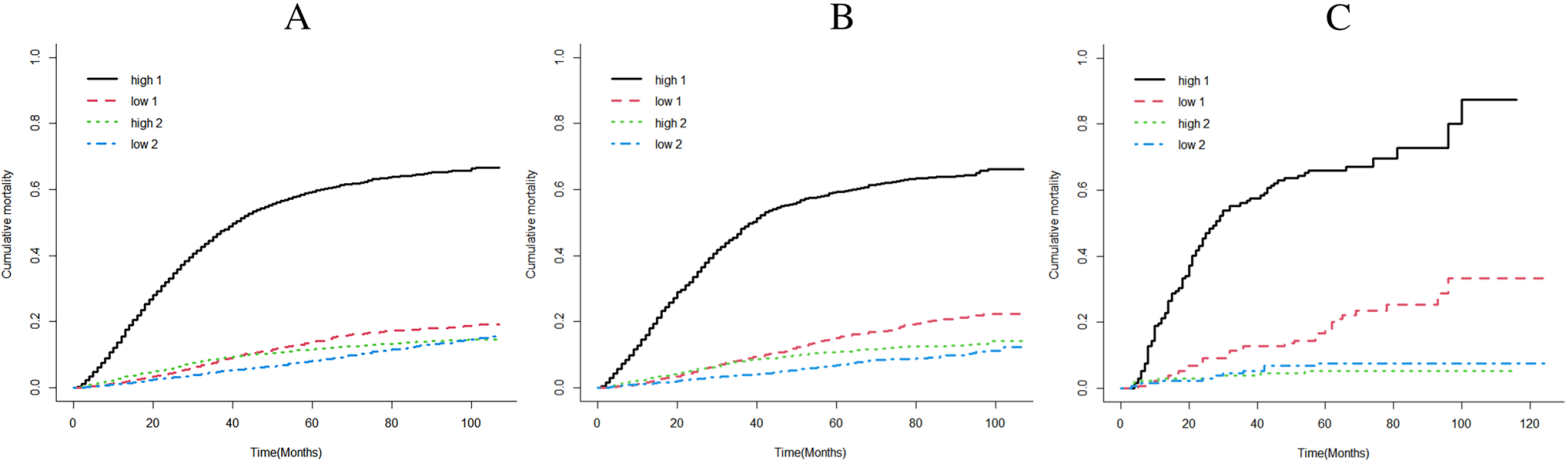
Estimated cumulative mortality of high-risk patients and low-risk patients in **A** training set, **B** internal validation set, and **C** external validation set. “1” represented cancer-specific death, and “2” represented death from other causes

We calculated the scores of each patient according to the prognosis-associated risk factors. In the training set, the lowest score was 38.84, the highest score was 472.79, and the median was 279.54. Taking the median score as the cut-off, the patients were classified into a low-risk group and a high-risk group, and performed risk stratification for data in both the internal validation set and the external one. Patients in the high-risk group had an evidently lower cumulative cancer-specific mortality than those in the low-risk group (Fig. 6A–C). The 1-year, 3-year, and 5-year cancer-specific mortality of the patients in the training set were 15.5%, 46.5%, and 59.4%, respectively, in the high-risk group, and were 1.6%, 8.0%, and 13.8%, respectively, in the low-risk group. The cancer-specific mortality of the patients in the validation set was similar to those in the training set.

### Comparison of different models for survival risk prediction

In addition, we compared the competitive risk model with conventional survival analysis in the prediction of the mortality risk at different time points, as shown in Table 3. For data from SEER, the 1-year, 3-year, and 5-year risk of death were 9.61%, 30.69%, and 41.18%, respectively, by conventional survival analysis, and were 8.61%, 27.46%, and 36.72%, respectively, by the competitive risk model. As for data from our hospital, the 1-year, 3-year, and 5-year risk of death were 12.96%, 36.84%, 44.56%, respectively, by conventional survival analysis, and were 12.12%, 34.47%, 41.69%, respectively, by the competitive risk model.

**Table 3:** Differences between conventional survival analysis and the competitive risk model in predicting the risk of death at different time-points

SEER database				Our own data		
Time (month)	Mortality of conventional survival analysis (%)	Competitive risk model		Mortality of conventional survival analysis (%)	Competitive risk model	
		Cancer-specific mortality (%)	Mortality from other causes (%)		Cancer-specific mortality (%)	Mortality from other causes (%)
12	9.61	8.61	2.06	12.96	12.12	2.65
24	20.95	18.75	4.26	28.81	26.52	3.03
36	30.69	27.46	6.50	36.84	34.47	4.55
60	41.18	36.72	9.60	44.56	41.69	6.44

## Discussion

In this study, we used a competitive risk model based on SEER and introduced a nomogram that could predict the 1-year, 3-year, and 5-year CSS of intermediate and advanced colon cancer patients who had undergone surgery and chemotherapy, and assessed its performance through internal and external validation. The c-index, ROC, and calibration curve indicated that the nomogram constructed in this study was of great reliability and remarkable predictive value, and its predictive performance was significantly better than the AJCC-TNM staging system. Furthermore, we performed risk stratification in those patients. The cancer-specific mortality of high-risk patients was higher than those with low risk at different time points, which could be helpful to the clinical risk-stratifying and decision-making.

We found that the 1-year, 3-year, and 5-year CSS predicted by the conventional survival analysis were lower than that predicted by the competitive risk model, which suggests bias in the results of conventional survival analysis under the existence of competitive risk (Wolbers et al. 2014) so that the mortality of colon cancer patients could be overestimated. It is estimated that about 46% of medical studies using the Kaplan–Meier method for survival analysis are affected by competing risk factors, which may lead to an overestimation of the real risk of the event by 10% (Walraven and McAlister 2016). A study by Zhou et al. (2019) yielded a consistent conclusion. If it does not be taken into account the effects of competitive risk events on the prognosis, there would be an underestimated survival of colon cancer patients at stage—I and -III. On the other hand, there are studies proposing that a competitive risk model is more proper to be recommended in the assessment of disease-specific mortality. It is of higher reliability and more accurate predictive value (Wolbers et al. 2014; Verduijn et al. 2011; Xu et al. 2021a). TNM-staging system remains the most commonly used tool in clinical settings for the prognostic assessment of cancer patients, which assesses the patients` prognosis mainly based on tumor size and depth of invasion (T stage), local lymph nodes involvement (N stage), and distant metastasis (M stage), and has been demonstrated to be applicable for the whole population (Xu et al. 2021a). However, the TNM-staging system has its own limitations in precisely distinguishing individual prognostic variances. Except for TNM-staging, several demographical or clinical information, such as age, marital status, treatments, and tumor biomarkers, could also affect patients` prognosis (Liu et al. 2020; Xu et al. 2021b). In recent years, nomogram presents to be of great potential in the field of oncology, which forms, by adding other prognosis-associated key factors on the basis of AJCC-TNM staging, a comprehensive clinical predictive model (Balachandran et al. 2015), and lists variables in the form of figures. By quantifying each variable into a specific score, it calculates the cumulative score of all the variables and matches it with the result scale to produce the final predicted probability (Wang et al. 2020). Nomograms can help clinicians and patients to make more accurate decisions due to their merits of being intuitive, individualized, and rapid in prognostic prediction (Balachandran et al. 2015; Li et al. 2021).

In this study, 18 independent risk factors were identified to be associated with the prognosis of intermediate and advanced colon cancer patients receiving surgery and chemotherapy, and constructed a comprehensive prediction model with these factors in the form of the nomogram. We found that age was evidently associated with the survival of colon cancer patients. The older the patient was, the worse the prognosis would be, which might be related to progressive organ failure and increasing susceptibility to concomitant diseases (Yamano et al. 1990; Sorbye et al. 2013). CEA level is also a crucial indicator revealing the prognosis of colon cancer patients (Locker et al. 2006). CEA testing is a necessary preoperative examination. A study by Zhou et al. (2019) discovered that stage-I, -II, and -III colon cancer patients with higher CEA levels had significantly poorer OS and CSS. In this study, we also observed that the prognosis of stage-III and -IV colon patients with higher CEA levels was poorer. We also found that a larger tumor size was associated with a poorer prognosis, which could be related to that large tumors tends to have stronger invasiveness. Another study Saha et al. (2015) based on U.S. National Cancer Database also found that among colon cancer patients, a larger tumor size indicated a poorer prognosis. Moreover, the number of lymphatic metastases is an important risk factor for assessing the prognosis of colon cancer patients. More lymph node metastases would indicate poorer survival outcomes (Kataoka et al. 2019). We focused not just on the number of positive lymph nodes because the results were subjected to the number of lymph nodes submitted for examination during the surgery. We used LNR as an alternative, and it has been demonstrated that LNR is more accurate than the N stage in predicting the prognosis of the patients (Parnaby et al. 2015). In addition, we assessed the effects of tumor deposition and perineural invasion on the patients` prognosis. According to the definition in the 7th edition of the AJCC-TNM classification, tumor deposition, also known as cancer nodules, refers to isolated nodules located within the lymphatic draining area of the primary lesion. These nodules often contain no identifiable lymph nodes, blood vessels, or nerves. The presence of tumor deposition has been proven to be a prognosis-associated risk factor colon cancer patients (Cohen et al. 2021). This is consistent with the results shown by the nomogram in this study. Multiple studies (Mirkin et al. 2018; Zheng et al. 2020) have found that colon cancer patient concomitant with tumor deposition and lymph node metastasis could have poorer survival outcomes. Perineural invasion refers to the invasion of cancer cells into nerve tissues surrounding the intestinal wall, which reflects the histopathological characteristics of tumor invasion and is considered to be an indicator of adverse prognosis in colon cancer patients (Skancke et al. 2019). Mayo et al. (2016) reported that patients with perineural invasion would have a more adverse prognosis. Perineural invasion could directly affect the OS and CSS of colon cancer patients, which is consistent with what we observed in this study. Besides, other independent risk factors such as marital status, gender, history of metastasectomy, and tumor grades have been demonstrated by multiple studies (Jin et al. 2020; Kuai et al. 2021; Lv et al. 2021b).

This is the first study using a competitive risk model for CSS-prediction in intermediate and advanced colon cancer patients after receiving surgery and chemotherapy. It is demonstrated, through internal and external validation, that the model is of considerable accuracy and reliability. However, some limitations still exist. Firstly, the SEER database cannot include all the risk factors that are associated with the patients' prognosis, which could result in selection bias when modeling. Targeted therapy is another crucial approach for colon cancer patients at intermediate and advanced stages, especially for those at stage-IV and with metastasis. Secondly, the data in the SEER database are incomplete. For example, it only collects the patients' CEA levels tested before the surgery, while the postoperative and follow-up results are not included. Postoperative CEA level would be more instructive (Konishi et al. 2018). Patients with a CEA level that increased before the surgery and decreased to normal after the surgery have a similar prognosis as those with a CEA level normal before the surgery, while patients with a postoperative CEA level still over the normal baseline might have a higher risk of local recurrence and metastasis. On the other hand, there are no records of specific regimens and courses for patients who received chemotherapy. Lastly, this study is a retrospective-design, and further validation is needed in prospective populations.

## Conclusions

Based on data from the SEER database and our hospital, we have successfully constructed the first predictive model for the CSS of intermediate and advanced colon cancer patients after receiving surgery and chemotherapy. The model has shown to be of great predictive performance in both the training set and the validation set, and is more effective than the AJCC-TNM staging system. It can help clinicians to make individualized treatment and follow-up strategies.

## Supplementary Information

Below is the link to the electronic supplementary material.

Supplementary file1 (DOCX 15 KB)

Supplementary file2 (DOCX 15 KB)

Supplementary file3 (CSV 266626 KB)

## Data Availability

The raw data supporting the conclusions of this article will be made available by the authors, without undue reservation.
