# Supplementary material for: Construction and validation of a prognostic nomogram for predicting cancer-specific survival in patients with intermediate and advanced colon cancer after receiving surgery and chemotherapy
Source: J Cancer Res Clin Oncol. 2023 Jul 17;149(14):12821–34. doi: 10.1007/s00432-023-05154-7 (PMC10587224; doi:10.1007/s00432-023-05154-7)
Supplement: Supplementary file 1 — Supplementary file1 (DOCX 15 KB) [file 432_2023_5154_MOESM1_ESM.docx]

**Construction and validation of a prognostic nomogram for predicting cancer-specific survival in patients with intermediate and advanced colon cancer after receiving surgery and chemotherapy**

Yiheng Shi^1,2#^, Xiaoting Wu^2#^, Wanxi Qu^1#^, Jiahao Tian^1^, Xunlei Pang^2^, HaoHan Fan^1^, Sujuan Fei^2,4*^, Bei Miao^3,4*^

^1^First Clinical Medical College, Xuzhou Medical University, Xuzhou Jiangsu 221002, China

^2^Department of Gastroenterology, The Affiliated Hospital of Xuzhou Medical University, Xuzhou Jiangsu 221002, China

^3^Institute of Gastroenterology, Xuzhou Medical University, Xuzhou Jiangsu 221004, China

^4^Key Laboratory of Gastrointestinal Endoscopy, Xuzhou Medical University, Xuzhou, Jiangsu, 221002, China

**^*^Corresponding author:** **Sujuan Fei**, Department of Gastroenterology, The Affiliated Hospital of Xuzhou Medical University, 99 West Huaihai Road, Xuzhou, Jiangsu Province, 221002, China; Key Laboratory of Gastrointestinal Endoscopy, Xuzhou Medical University, Xuzhou, Jiangsu, 221002, China. Email: [xyfyfeisj99@163.com](mailto:xyfyfeisj99@163.com) ;

**ORCID:** 0000-0002-7828-028X

**Bei Miao**, Institute of Digestive Diseases, Xuzhou Medical University, 84 West Huaihai Road, Xuzhou, Jiangsu Province, 221002, China; Key Laboratory of Gastrointestinal Endoscopy, Xuzhou Medical University, Xuzhou, Jiangsu,221002, China. Email: miaobei@xzhmu.edu.cn.

Supplementary Table 1 The detailed selection process of patients

| Excluded Variables | Remove reasons | Exclude sample size | Remaining sample size | Total sample size |
| --- | --- | --- | --- | --- |
| Derived AJCC Stage Group, 7th ed (2010-2015) | 0、I、II、IIA、IIb、IIc、IInos、NA、UNK | 6659 | 33447 | 40106 |
| Survival months | 0 | 15 | 33432 |  |
| SEER cause-specific death classification | Dead (missing/unknown COD) | 221 | 33211 |  |
| Site recode ICD-O-3/WHO 2008 | Large Intestine, NOS、Appendix | 1920 | 31291 |  |
| Race recode (W, B, AI, API) | Unknown | 78 | 31213 |  |
| Grade (thru 2017) | Unknown | 900 | 30313 |  |
| Derived AJCC T, 7th ed (2010-2015) | Tx | 192 | 30121 |  |
| Derived AJCC N, 7th ed (2010-2015) | Nx | 32 | 30089 |  |
| RX Summ--Surg Prim Site (1998+) | 99 | 5 | 30084 |  |
| RX Summ--Scope Reg LN Sur (2003+) | Number of regional lymph nodes removed unknown、Sentinel node biopsy and lym nd removed same/unstated time、Biopsy or aspiration of regional lymph node, NOS、Sentinel lymph node biopsy、Sentinel node biopsy and lym nd removed different times | 302 | 29782 |  |
| RX Summ--Surg Oth Reg/Dis (2003+) | Unknown; death certificate only | 16 | 29766 |  |
| Radiation recode | Recommended, unknown if administered | 107 | 29659 |  |
| CEA Pretreatment Interpretation Recode (2010+) | Not documented; Interpretation not assessed or unknown if assessed、Test ordered, results not in chart、Borderline、 | 9591 | 20068 |  |
| Perineural Invasion Recode (2010+) | Not documented/assessed; No mention in path report; Pathologist cant determine | 1461 | 18607 |  |
| Tumor Deposits Recode (2010+) | Not documented/assessed; Indeterminate; No mention in path report; No resection | 641 | 17966 |  |
| Regional nodes positive (1988+) | 95/97/98/99 | 97 | 17869 |  |
| SEER Combined Mets at DX-bone (2010+) | N/A、unknown | 95 | 17774 |  |
| SEER Combined Mets at DX-brain (2010+) | N/A、unknown | 19 | 17755 |  |
| SEER Combined Mets at DX-liver (2010+) | N/A、unknown | 21 | 17734 |  |
| SEER Combined Mets at DX-lung (2010+) | N/A、unknown | 30 | 17704 |  |
| CS tumor size (2004-2015) | 990/998/999 | 386 | 17318 |  |
| Marital status at diagnosis | Unknown | 683 | 16635 |  |
| Regional nodes examined (1988+) | 96/97/98 | 14 | 16621 |  |
