# Supplementary material for: Construction and validation of a prognostic nomogram for predicting cancer-specific survival in patients with intermediate and advanced colon cancer after receiving surgery and chemotherapy
Source: J Cancer Res Clin Oncol. 2023 Jul 17;149(14):12821–34. doi: 10.1007/s00432-023-05154-7 (PMC10587224; doi:10.1007/s00432-023-05154-7)
Supplement: Supplementary file 2 — Supplementary file2 (DOCX 15 KB) [file 432_2023_5154_MOESM2_ESM.docx]

**Construction and validation of a prognostic nomogram for predicting cancer-specific survival in patients with intermediate and advanced colon cancer after receiving surgery and chemotherapy**

Yiheng Shi^1,2#^, Xiaoting Wu^2#^, Wanxi Qu^1#^, Jiahao Tian^1^, Xunlei Pang^2^, HaoHan Fan^1^, Sujuan Fei^2,4*^, Bei Miao^3,4*^

^1^First Clinical Medical College, Xuzhou Medical University, Xuzhou Jiangsu 221002, China

^2^Department of Gastroenterology, The Affiliated Hospital of Xuzhou Medical University, Xuzhou Jiangsu 221002, China

^3^Institute of Gastroenterology, Xuzhou Medical University, Xuzhou Jiangsu 221004, China

^4^Key Laboratory of Gastrointestinal Endoscopy, Xuzhou Medical University, Xuzhou, Jiangsu, 221002, China

**^*^Corresponding author:** **Sujuan Fei**, Department of Gastroenterology, The Affiliated Hospital of Xuzhou Medical University, 99 West Huaihai Road, Xuzhou, Jiangsu Province, 221002, China; Key Laboratory of Gastrointestinal Endoscopy, Xuzhou Medical University, Xuzhou, Jiangsu, 221002, China. Email: [xyfyfeisj99@163.com](mailto:xyfyfeisj99@163.com) ;

**ORCID:** 0000-0002-7828-028X

**Bei Miao**, Institute of Digestive Diseases, Xuzhou Medical University, 84 West Huaihai Road, Xuzhou, Jiangsu Province, 221002, China; Key Laboratory of Gastrointestinal Endoscopy, Xuzhou Medical University, Xuzhou, Jiangsu,221002, China. Email: miaobei@xzhmu.edu.cn.

Supplementary Table 2 Rename and define the factors

| factors | values | define |
| --- | --- | --- |
| Sex | 1 | Male |
| Sex | 2 | Female |
| Race | 0 | White |
| Race | 1 | Black |
| Race | 2 | Other |
| Marital_status | 0 | Married |
| Marital_status | 1 | Single |
| Marital_status | 2 | Other |
| Tumor_site | 1 | Ascending Colon |
| Tumor_site | 2 | Cecum |
| Tumor_site | 3 | Descending Colon |
| Tumor_site | 4 | Hepatic Flexure |
| Tumor_site | 5 | Sigmoid Colon |
| Tumor_site | 6 | Splenic Flexure |
| Tumor_site | 7 | Transverse Colon |
| Tumor_grade | 1 | GradeI/II |
| Tumor_grade | 2 | GradeIII/IV |
| Tumor_stage | 1 | III |
| Tumor_stage | 2 | IV |
| T_stage | 1 | T1-2 |
| T_stage | 2 | T3 |
| T_stage | 3 | T4 |
| N_stage | 1 | NO/N1 |
| N_stage | 2 | N2 |
| Surg_type | 1 | Partial colectomy |
| Surg_type | 2 | Subtotal colectomy |
| Surg_type | 3 | Total colectomy |
| Surg_type | 4 | Other |
| Lymphadenectomy | 0 | None |
| Lymphadenectomy | 1 | January 3rd |
| Lymphadenectomy | 2 | ≥4 |
| Metastasectomy | 0 | NO |
| Metastasectomy | 1 | YES |
| Radiation | 0 | No/Unknown |
| Radiation | 1 | YES |
| CEA | 0 | normal |
| CEA | 1 | positive |
| Tumor_Deposits | 0 | NO |
| Tumor_Deposits | 1 | YES |
| Perineural_Invasion | 0 | Not identified |
| Perineural_Invasion | 1 | Identified |
| Bone_metastasis | 0 | NO |
| Bone_metastasis | 1 | YES |
| Brain_metastasis | 0 | NO |
| Brain_metastasis | 1 | YES |
| Liver_metastasis | 0 | NO |
| Liver_metastasis | 1 | YES |
| Lung_metastasis | 0 | NO |
| Lung_metastasis | 1 | YES |
| First_malignant | 0 | NO |
| First_malignant | 1 | YES |
